# Age-related sensory decline mediates the Sound-Induced Flash Illusion: Evidence for reliability weighting models of multisensory perception

**DOI:** 10.1038/s41598-019-55901-5

**Published:** 2019-12-18

**Authors:** Rebecca J. Hirst, Annalisa Setti, Rose A. Kenny, Fiona N. Newell

**Affiliations:** 1 School of Psychology and Institute of Neuroscience, Trinity College Dublin, Dublin, Ireland; 2 The Irish Longitudinal Study on Ageing, Trinity College Dublin, Dublin, Ireland; 3 School of Applied Psychology, University College Cork, Dublin, Ireland; 4 Mercer’s Institute for Successful Ageing, St. James Hospital, Dublin, Ireland

**Keywords:** Sensory processing, Human behaviour

## Abstract

Perception of our world is proposed to arise from combining multiple sensory inputs according to their relative reliability. We tested multisensory processes in a large sample of 2920 older adults to assess whether sensory ability mediates age-related changes in perception. Participants completed a test of audio-visual integration, the Sound Induced Flash Illusion (SIFI), alongside measures of visual (acuity, contrast sensitivity, self-reported vision and visual temporal discrimination (VTD)) and auditory (self-reported hearing and auditory temporal discrimination (ATD)) function. Structural equation modelling showed that SIFI susceptibility increased with age. This was mediated by visual acuity and self-reported hearing: better scores on these measures predicted reduced and stronger SIFI susceptibility, respectively. Unexpectedly, VTD improved with age and predicted increased SIFI susceptibility. Importantly, the relationship between age and SIFI susceptibility remained significant, even when considering mediators. A second model showed that, with age, visual ‘gain’ (the benefit of congruent auditory information on visual judgements) was predicted by ATD: better ATD predicted stronger visual gain. However, neither age nor SIFI susceptibility were directly associated with visual gain. Our findings illustrate, in the largest sample of older adults to date, how multisensory perception is influenced, but not fully accounted for, by age-related changes in unisensory abilities.

## Introduction

Sensory decline is a normal part of the ageing process^1,2^. However, age-related loss in one sensory modality does not necessarily correspond to loss in other senses^3^, resulting in fluctuations in the balance of sensory abilities across the lifespan. It is therefore not surprising that the way in which we combine inputs from several senses, *multisensory integration*, is also influenced by ageing. Experimental evidence suggests that older adults show increased multisensory integration relative to younger adults. For example, in the Sound Induced Flash Illusion (SIFI), the presentation of a single visual flash with two beeps results in the illusory perception of two flashes^4^. Older adults appear more susceptible to this illusion^5,6^ and remain susceptible over wider ranges of stimulus-onset asynchrony (SOA)^6,7^, in line with an age-related widening of the temporal binding window^8^ (also known as the Temporal Window of Integration^9^). Increased multisensory integration in ageing might reflect a compensatory mechanism, serving to increase the likelihood of detecting important stimuli and events in the environment following unisensory decline^10–12^ (for reviews see^13,14^).

Current models of multisensory perception propose that our unified multisensory percept results from weighting unisensory inputs according to their reliability. These accounts include the *information reliability hypothesis*^15^, *Maximum Likelihood Estimation*^16,17^ and *Bayesian inference* models^18^, whereby information from each sense is weighted according to its relative reliability and precision in a given task, based upon the quality of current input and, in Bayesian models, our prior knowledge about the world^19^. This prior knowledge may take the form of a “coupling prior” whereby knowledge regarding the likelihood of a common source between two sensory signals is used to optimally integrate information^17,20^. Given this, age-related unisensory decline should affect integration by lowering the reliability of sensory input, as well as our prior knowledge about the usefulness of each sense. Indeed, it has been proposed that perception in older adults is associated with increased reliance on priors when making perceptual judgements^21^.

Susceptibility to the SIFI is thought to follow the rules of optimal integration^19^. As such, because audition typically provides more accurate perception regarding temporal judgements, sound influences the perception of visual temporal events. In addition to top-down knowledge regarding the usefulness of each sense in a given task, the quality of sensory input also influences reliability weighting. For example, lowering the stimulus contrast or blurring the visual image reduces susceptibility to illusions in which vision typically dominates, such as the ventriloquist^22^ and the McGurk^23^ effect. In the SIFI, lowering the reliability of vision relative to audition (for example through age-related decline in acuity) should increase SIFI susceptibility, whilst lowering the reliability of audition relative to vision (for example through hearing loss) should lower SIFI susceptibility. Furthermore, in line with compensatory accounts of multisensory integration in ageing^10–14^, and the superadditive properties of multisensory neurones (i.e. proportionally larger firing rates from multisensory neurones when unisensory signals are weak^24^), older adults experiencing stronger visual decline (and weaker visual input) would be expected to benefit from multisensory stimulation. For example, it might be expected that older adults would show better performance for judging two flashes presented with two sounds compared with judging the flashes presented alone.

Despite the theoretical plausibility of this hypothesis, studies that have manipulated the relative visibility or audibility of stimuli in the SIFI, and clinical studies with individuals with sensory impairment, have found mixed results. On the one hand, Shams *et al*. (2000) found that susceptibility to the SIFI was not influenced by changes in visual contrast. However, increasing the luminance of the visual stimulus increased SIFI susceptibility in young adults^25^. On the other hand, in line with reliability models, lowering the intensity of the auditory stimulus to near-threshold levels can produce reverse SIFI effects, such that the flash alters the perception of the beep^26^. In young adults, SIFI susceptibility also appears greatest when auditory stimuli are presented over headphones^27^, perhaps due to a reduction in ambient noise and increased certainty regarding the occurrence of auditory stimuli. Patients with amblyopia, a neurodevelopmental condition affecting visual acuity and contrast sensitivity, also manifest stronger SIFI effects over wider time windows^28^. Furthermore, older adults with mild hearing loss have reduced SIFI susceptibility, which seems to be restored to age normal levels through hearing aid use^29^. Evidence from these studies therefore lends support to the hypothesis that age-related changes in unisensory reliability should influence multisensory perception. However, whether different facets of sensory processing differentially influence SIFI susceptibility remain unclear. For example, contrast sensitivity might not influence^4^ or, counter intuitively, increase^25^ SIFI susceptibility, whilst other factors such as mild hearing impairment^29^ and poorer visual acuity^28^ might decrease and increase SIFI susceptibility respectively. Therefore it is necessary to test whether the information reliability hypothesis holds for the SIFI in the ageing population.

In this study, we explored the role of age-related sensory change on multisensory integration in the largest sample of older adults to date. We considered several aspects of sensory function including visual acuity, contrast sensitivity and self-reported ability in vision and audition. We also considered temporal discrimination abilities in vision and audition, by utilising performance for judging two flashes and two beeps presented unimodally. In contrast to what would be predicted based on reliability models, increased visual temporal resolution has been associated with stronger SIFI susceptibility^25^. However, the study reported by Pérez-Bellido *et al*., (2015) focused on young adults and did not assess auditory temporal resolution in a similar way. Our study therefore allowed us to distinguish between the role of acuity, contrast sensitivity and temporal abilities in both vision and audition within a large sample of 2920 individuals aged from 50 years old.

To our knowledge, no study has investigated the effect of age-related decline in unisensory acuity and temporal discrimination abilities on multisensory integration within a large, representative, sample of individuals aged over 50 years. This is despite the common and high occurrence of age-related sensory loss as well as recent reviews suggesting that individual differences in unisensory function might account for heterogeneous findings regarding multisensory integration in ageing^30^. Furthermore, given the increased popularity of SIFI as a measure of multisensory integration, the clinical relevance of assessing integration in ageing^6,31^ and experimental evidence showing that multisensory integration can be trained^32^, an understanding how the SIFI is affected by sensory decline is of central importance in the interpretation of SIFI as an outcome measure.

The goal of the current study was, therefore, to investigate the impact of unisensory function upon multisensory integration in ageing. We used data from The Irish Longitudinal Study of Ageing (TILDA), a study of a representative sample of community dwelling older adults in the Republic of Ireland. In 2014, TILDA became the first longitudinal study of ageing to include an assessment of multisensory integration (i.e. a task based on the SIFI) as part of its health assessment. In addition to this, TILDA also gathers several measures of visual function (contrast sensitivity, acuity and self-reported vision) and self-reported hearing measures. Thus, the available data allowed us, for the first time, to test whether reliability weighting models of multisensory integration are manifested in age-related sensory changes within a large cohort of individuals. The specific research questions we aimed to address were:

1. Is the relationship between age and SIFI susceptibility mediated by unisensory changes in vision and hearing?
2. Is the relationship between age and visual gain (i.e. better accuracy for judging two visual stimuli paired with two sounds versus vision alone) mediated by unisensory measures of vision and audition?
3. Does visual gain predict SIFI susceptibility?

We answered these questions using Structural Equation Modelling (SEM), in which measures of sensory function were considered as mediator variables for the effect of age upon the illusory (i.e. SIFI) and compensatory (i.e. visual gain) effects of multisensory integration. We hypothesised that ageing would result in both increased SIFI susceptibility and visual gain, and that these would be mediated by unisensory function. Reduced visual ability should predict stronger SIFI perception and gains on vision from congruent auditory information, whereas reduced auditory function should predict fewer SIFI illusions and less visual gain (i.e. a reduction in the influence of congruent auditory information on visual perception). We also hypothesised that greater visual gain would be associated with stronger SIFI susceptibility: that is, those who use audition to benefit vision should also be more susceptible to the illusory influence of sound.

## Results

### Sensory ability and relative reliability in relation to SIFI

**Figure 1:**
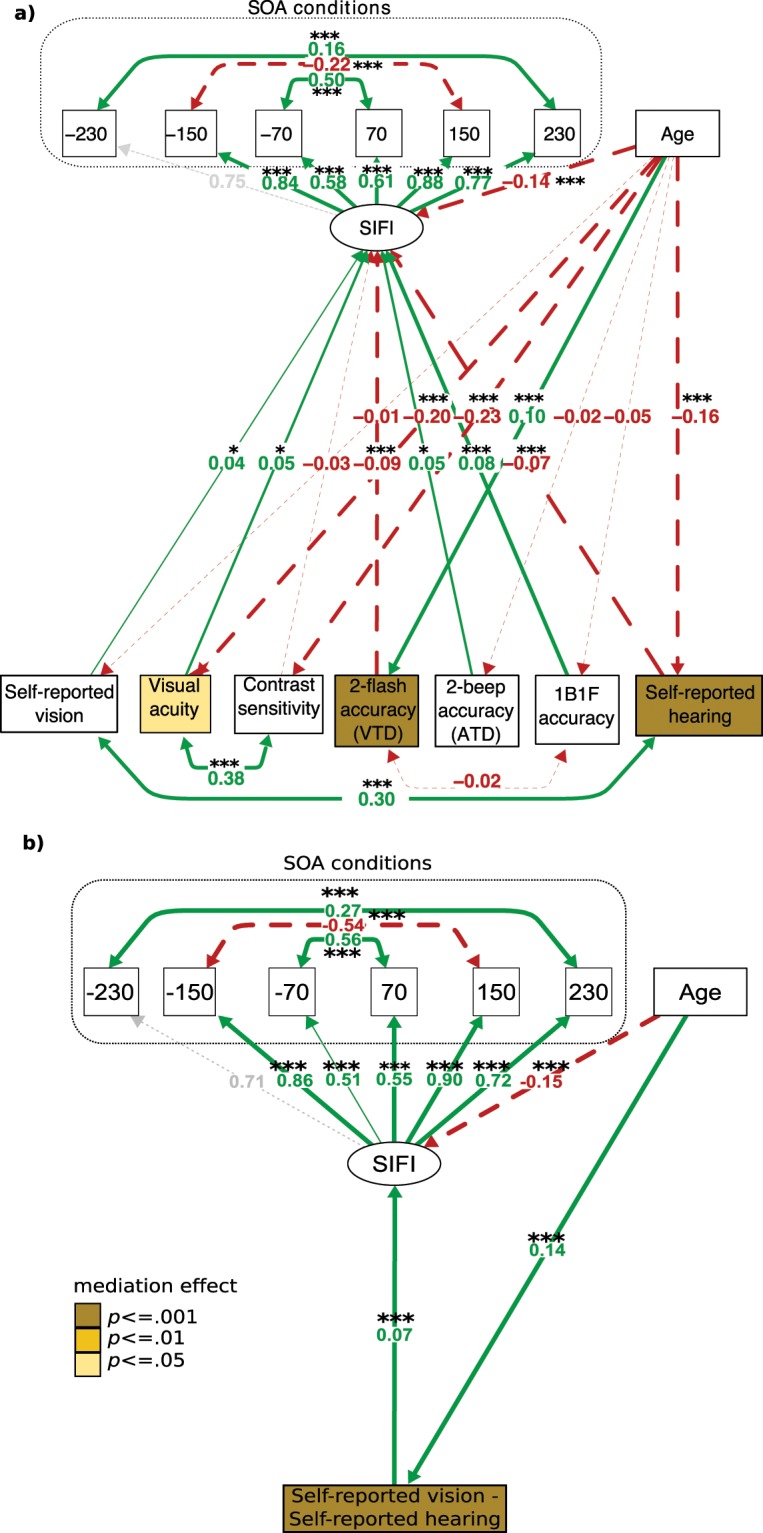
(**a**) Structural equation model (SEM) for model 1a and (**b**) SEM for model 1b. In 1b the mediator is the difference score between self-reported vision and self-reported hearing. ***p = < 0.001, **p < = 0.01, *p < = 0.05. Paths are labelled with standardised coefficients. Red dashed lines indicate negative coefficients, green continuous lines indicate positive coefficients, grey path indicates constrained path for confirmatory factor analysis. SIFI = accuracy for illusory 2B1F trials. The thickness of each line corresponds to the significance of the path. Contrast sensitivity reflects area under the contrast sensitivity curve. Visual acuity reflects “visual acuity score” – see methods.

Our first question was whether the relationship between age and SIFI was mediated by differences in sensory ability (model 1a) and the relative reliability of vision versus audition (model 1b). Note that SIFI susceptibility is determined by decreased accuracy for judging one flash when one flash is presented with two beeps (2B1F). Throughout our results section, “stronger SIFI susceptibility” therefore corresponds to reduced accuracy on illusory trials (i.e. the red dashed paths in Fig. 1) whilst “reduced SIFI susceptibility” corresponds to increased accuracy (i.e. the green continuous paths in Fig. 1). Table 1 illustrates the proportion correct for each SIFI condition stratified by age (age was continuous in the model). Figure 1 shows the resulting standardized coefficients for models 1a and 1b.

**Table 1:** Proportion correct for SIFI conditions stratified by age group.

			Age (years)					Age (years)		
			50–64	65–74	>=75			50–64	65–74	>=75
Non-illusory conditions	2B2F 70	Mean	0.57	0.57	0.53	0B2F (VTD)	Mean	0.21	0.23	0.23
		SD	0.4	0.41	0.42		SD	0.33	0.35	0.34
		Med.	0.5	0.5	0.5		Med.	0	0	0
	2B2F 150	Mean	0.88	0.89	0.89	2B0F (ATD)	Mean	0.59	0.55	0.5
		SD	0.26	0.26	0.27		SD	0.42	0.43	0.43
		Med.	1	1	1		Med.	0.5	0.5	0.5
	2B2F 230	Mean	0.94	0.93	0.93	1B1F	Mean	0.97	0.97	0.97
		SD	0.2	0.21	0.22		SD	0.14	0.14	0.14
		Med.	1	1	1		Med.	1	1	1
Illusory conditions	2B1F 70	Mean	0.53	0.51	0.5	2B1F -70	Mean	0.5	0.48	0.48
		SD	0.43	0.44	0.44		SD	0.44	0.44	0.45
		Med.	0.5	0.5	0.5		Med.	0.5	0.5	0.5
	2B1F 150	Mean	0.41	0.31	0.28	2B1F -150	Mean	0.31	0.24	0.22
		SD	0.43	0.41	0.41		SD	0.4	0.37	0.36
		Med.	0.5	0	0		Med.	0	0	0
	2B1F 230	Mean	0.46	0.33	0.24	2B1F -230	Mean	0.3	0.21	0.18
		SD	0.45	0.41	0.39		SD	0.4	0.36	0.34
		Med.	0.5	0	0		Med.	0	0	0

Note: age was entered as a continuous variable during modelling (see Fig. 4b for distribution). Each participant could score 0, 0.5 or 1 proportion correct per condition. Each SIFI condition is denoted by the number of beeps (“B”), number of flashes (“F”) and stimulus onset asynchrony (SOA) in ms (a negative value indicates that one beep was presented before the flash-beep pair).

For model 1a the RMSEA was initially 0.062 (CI = [0.58, 0.66], *pclose* < 0.001). Following modification indices, the covariance between self-reported vision and hearing was considered in the model, improving overall fit (RMSEA = 0.051 CI = [0.047, 0.055], *pclose* = 0.356). For model 1b RMSEA this indicated a close fit without the need for modification indices (RMSEA = 0.05, CI = [0.042, 0.058], *pclose* = 0.474). Here we outline these results and provide standardized coefficients (for the complete statistical results please see the Supplementary Materials).

The goal of model 1a was to assess whether sensory measures mediated the relationship between age and SIFI susceptibility. Age significantly predicted visual acuity, contrast sensitivity, visual temporal discrimination (VTD) and self-reported hearing. Performance on all of these measures, apart from VTD, decreased with increasing age. Age did not significantly predict self-reported vision or auditory temporal discrimination (ATD). Unexpectedly, increasing age was associated with better VTD and this was, in turn, associated with stronger SIFI susceptibility. To identify whether this result was based on a response bias (i.e. that participants were more likely to make a “2 flash” response, resulting in correct performance on the 0B2F and incorrect performance on the 2B1F condition), we included performance on the 1B1F condition as a mediator, and explored whether there was a a significant negative relationship between performance on the unimodal 0B2F condition and 1B1F condition: a response bias would predict higher accuracy in the 0B2F and lower accuracy in the 1B1F condition. In contrast to a response bias interpretation, however, this relationship failed to reach significance.

As predicted, ageing was associated with stronger SIFI susceptibility. Contrast sensitivity was the only sensory measure that did not predict SIFI susceptibility. In line with reliability weighting, better visual acuity and self-reported vision were associated with reduced SIFI susceptibility, and higher self-reported hearing ability was associated with greater SIFI susceptibility. However, in contrast to reliability weighting (and perhaps counter-intuitively), better ATD and VTD were associated with reduced and stronger SIFI susceptibility respectively.

To specifically answer our first research question, mediation analysis showed that three measures, visual acuity, self-reported hearing and VTD mediated the relationship between age and SIFI susceptibility. As described, the former two of these mediators followed the predicted pattern of effects, whereby better visual acuity resulted in weaker SIFI and better self-reported hearing resulted in stronger SIFI susceptibility. The third mediator, VTD, did not follow our predictions, as increased VTD predicted stronger SIFI susceptibility.

With regards to the relative reliability of vision versus audition (Fig. 1b), mediation analysis showed that the relationship between age and SIFI susceptibility was also mediated by the difference between self-reported vision and self-reported hearing. Age resulted in higher self-reported vision scores over audition and, in turn, this was associated with reduced SIFI susceptibility. However, as in model 1a, the relationship between age and SIFI susceptibility remained significant even when considering this mediator.

### Sensory ability and relative reliability in relation to visual gain

**Figure 2:**
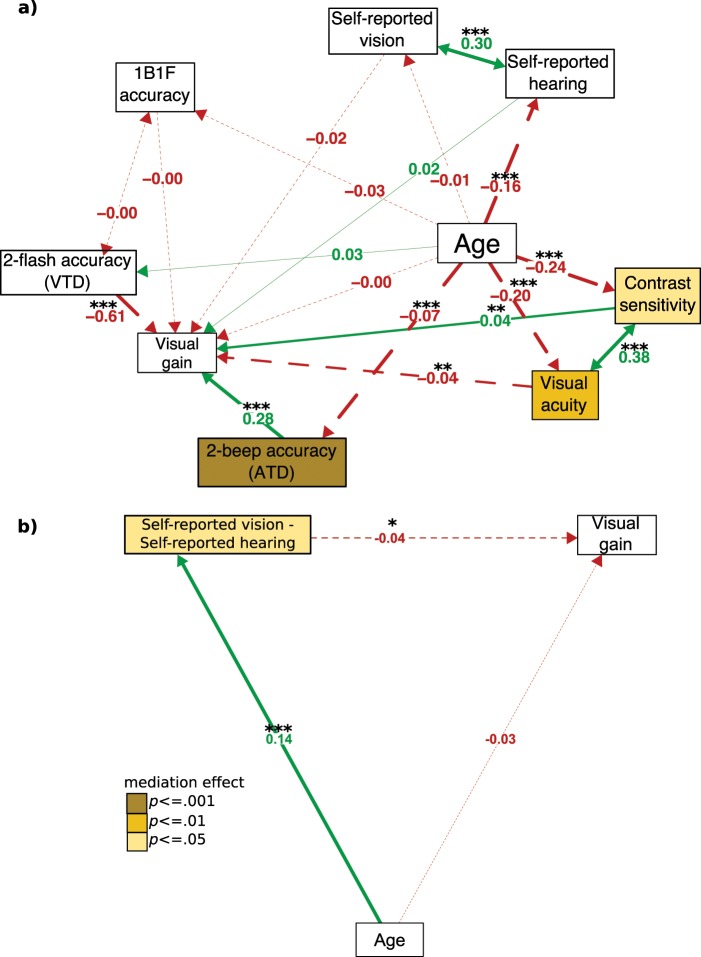
(**a**) Structural equation model (SEM) for model 2a and (**b**) SEM for model 2b. In 2b the mediator is the difference score between self-reported vision and self-reported hearing. ***p = < 0.001, **p < = 0.01, *p < = 0.05. Red dashed lines indicate negative coefficients green continuous lines indicate positive coefficients, grey path indicates constrained path for confirmatory factor analysis. Thickness of each line corresponds to its significance level. Contrast sensitivity reflects area under the contrast sensitivity curve. Visual acuity reflects “visual acuity score” – see methods.

Our second question was whether the relationship between age and visual gain was mediated by sensory ability (model 2a) and the reliability of vision over audition (model 2b). The results of these models are shown in Fig. 2. The initial RMSEA for model 2a suggested that modification indices would improve model fit (RMSEA = 0.079, CI = [0.072, 0.86], *pclose* < 0.001). As with model 1a, considering the covariance between self-reported vision and hearing improved the model fit (RMSEA = 0.042, CI = [0.033, 0.049], *pclose* = 0.960).

As in model 1a, age significantly predicted visual acuity, contrast sensitivity and self-reported hearing but not self-reported vision. In contrast to model 1a, the relationship between age and ATD but not VTD reached significance (although the directionality of effects remained the same). Age did not directly influence visual gain. However, better visual acuity and VTD were associated with lower visual gain. Better ATD predicted stronger visual gain. This is in line with the idea that gain would occur in individuals experiencing sensory loss and that more reliable ATD might benefit temporal visual judgements. However, contrary to this, better contrast sensitivity was also associated with more visual gain. Mediation analysis showed that ATD, visual acuity and contrast sensitivity mediated the relationship between age and visual gain.

With regards to relative reliability, model 2b again showed no significant relationship between age and visual gain. However, the performance of those whose self-reported vision was better than audition benefitted less from congruent auditory stimulation (i.e. less visual gain). In line with this, the mediation analysis revealed that the difference in self-reported vision and hearing was a significant mediator of the relationship between age and visual gain.

### The relationship between visual gain and SIFI susceptibility (Model 3)

**Figure 3:**
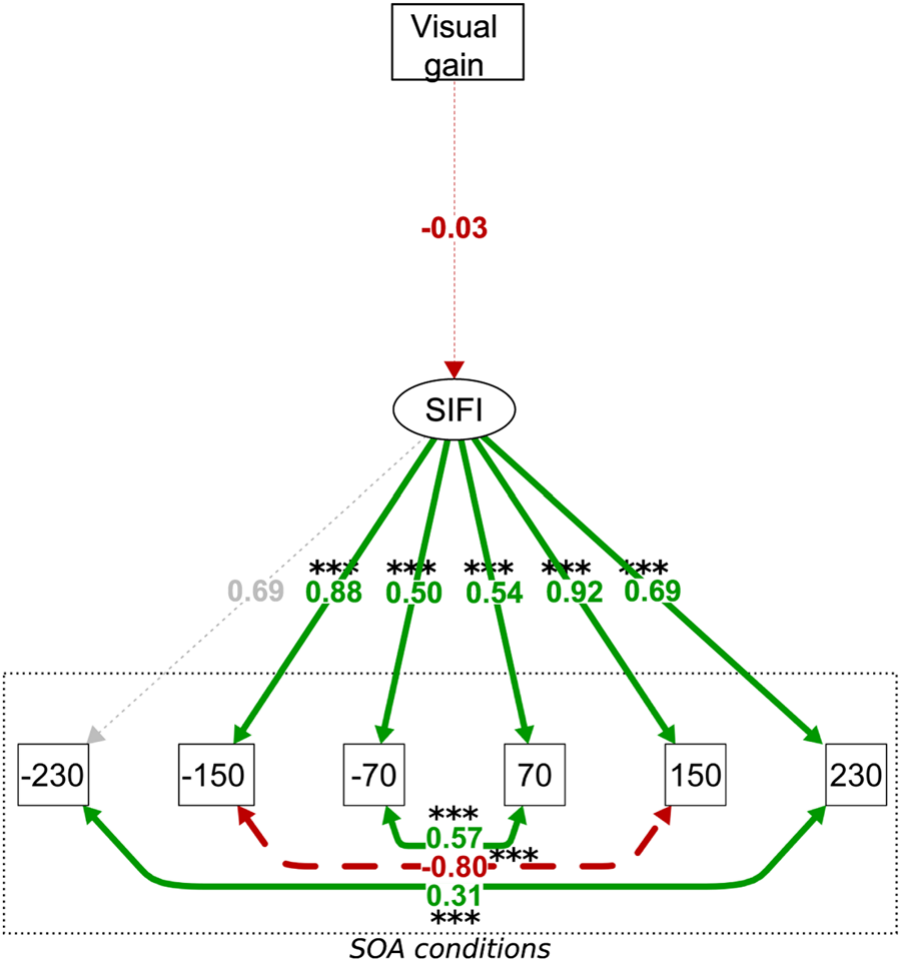
Structural equation model (SEM) for model 3; ***p = < 0.001, **p < = 0.01, *p < = 0.05. Red dashed paths indicate negative coefficients, green continuous paths indicate positive coefficients, grey path indicates constrained path for confirmatory factor analysis. Variance p values all significant < 0.001 and not shown. SIFI = accuracy for illusory 2B1F trials. The thickness of each line corresponds to its significance.

Our final question was whether visual gain predicted SIFI susceptibility. We hypothesised that participants manifesting stronger visual gain (i.e. those for whom congruent auditory information aided visual judgements) would be more susceptible to the SIFI. However, the relationship between visual gain and SIFI performance, although in the hypothesised direction, failed to reach significance (Fig. 3).

## Discussion

This study addressed whether age-related unisensory decline alters multisensory integration, in line with reliability weighting models of perception, within the largest cohort of older adults (n = 2920) tested to date. Multisensory integration was assessed using SIFI susceptibility and the extent to which accuracy for judging two flashes was enhanced by the congruent presentation of two beeps (which we termed “visual gain”). Visual acuity, self-reported hearing and the difference in self-reported vision and hearing mediated the relationship between age and SIFI susceptibility in a manner consistent with reliability weighting accounts of multisensory perception. However, the relationship between age and SIFI susceptibility remained significant even when considering mediators, thus, not all age-related changes appear to be accounted for by unisensory decline. With regards to visual gain, age did not directly predict gain. However, better visual function (acuity) predicted lower gain whereas better hearing function (ATD) predicted stronger gains, as expected. Thus, visual decline coupled with good auditory temporal discriminability may increase the likelihood of congruent auditory information facilitating visual temporal judgements. Finally, in contrast to our hypotheses, the relationship between visual gain and SIFI susceptibility failed to reach significance. Here, we expand upon several unexpected, but explainable results, consider the limitations of this study, and pose future directions.

### Increased visual temporal discrimination in ageing

Ageing was associated with increased accuracy for judging two flashes with 70 ms SOA (i.e. better VTD). This was unexpected, given known age-related declines in visual and auditory gap detection^33^. In turn, better VTD was associated with increased SIFI susceptibility. This was also unexpected based upon reliability weighting^19^. Improved VTD with age did not appear associated with response bias, as correct performance on the unimodal 2-flash condition was not significantly associated with incorrect performance on the 1B1F condition. This is consistent with findings that increased SIFI susceptibility with age is associated with changes in perceptual sensitivity but not response bias^7^ (but see also^34^).

Although unexpected, our findings are consistent with other interesting results in the SIFI and ageing literature, as well as other aspects of our model. Firstly, increased visual temporal resolution has previously been associated with stronger SIFI susceptibility^25^, although in contrast to reliability weighting, our results support this finding. Secondly, if we consider the two types of illusory percepts within the SIFI literature, “fission” illusions (i.e. perceiving 1 flash as 2 when presented with 2 beeps, as in our design) and “fusion” illusions (perceiving 2 flashes as 1 when presented with 1 beep), our findings might explain why older adults reportedly show increased fission but not fusion effects^7^. If older adults can accurately perceive 2 flashes, we would expect a weaker influence of incongruent auditory information (i.e. less fusion). Similarly, although we predicted increased visual gain with ageing, if older adults are good at judging 2 flashes, we would expect less influence from audition on vision (less gain), as was observed. It is possible that visual gain would be expected primarily in older adults manifesting low reliability of both vision and hearing, due to inverse effectiveness (see^35^ for discussion on inverse effectiveness).

One point to consider is that vision appears to be subjectively perceived as the more reliable modality in older adults. In model 1a ageing predicted reduced self-reported hearing, but not vision, and in model 1b increasing age resulted in better self-reported vision relative to hearing. In general, these findings are consistent with vision treated as the more reliable modality, relative to hearing, in ageing. It remains an open question whether a) older adults explicitly learn to rely on a sensory modality they self-report as better, b) genuine biological changes during ageing enable better visual temporal discrimination, or both.

### Differing effects of contrast sensitivity on multisensory integration and gain

Across models, contrast sensitivity had differing effects compared with other measures of visual function (i.e. acuity). Contrast sensitivity did not influence SIFI (model 1a) and better contrast sensitivity predicted stronger visual gain (model 1b). It is possible that contrast sensitivity was not associated with SIFI susceptibility because a high contrast stimulus was always presented, thus masking potential effects. However, previous research shows that adjusting the contrast of the visual stimulus does not alter SIFI susceptibility in young adults^4^, although it has also been reported that increasing luminance contrast can counter-intuitively increase SIFI susceptibility^25^. The positive relationship between contrast and visual gain also contradicts the hypothesis of compensatory multisensory integration in ageing, whereby stronger gains should be observed in those with poorer sensory function (although other sensory measures such as acuity and VTD support this compensatory hypothesis in our model).

One explanation is that contrast sensitivity and visual acuity reflect different neural functions, one of which might be more closely associated with the SIFI. Within the retino-cortico pathways, the magnocellular pathway (M-path) processes stimuli with close-to-threshold contrast and low spatial frequency, the parvocellular pathway (P-path) processes high contrast stimuli with high spatial frequency^36^. Lesions to the P-pathway but not the M-pathway reduce acuity in monkeys^37^. Although stimuli in the SIFI are high contrast, evidence suggests the SIFI is associated with processing in the M-pathway^25^. For example, the M-pathway manifests transient responses^38^ and is more strongly represented in the periphery^39^: in the SIFI task, visual stimuli are presented both transiently and peripherally. Paradoxically, contrast sensitivity and the SIFI should therefore share similar retino-cortico mechanisms and, as M and P subdivisions are maintained in the cortex^40^, similar cortical mechanisms. One possible explanation is that ageing might have a stronger effect on the contrast sensitivity of neurones at progressively higher stages of the visual pathway^41^. If the SIFI occurs at relatively early stages of visual processing (i.e. V1^42–44^) it is possible that reduced contrast sensitivity in ageing reflects deterioration at higher order stages than the level at which SIFI occurs. However, at present, this remains speculative and further research is required to tease these possibilities apart. The current findings highlight the importance of considering different measures of unisensory function in relation to multisensory integration in ageing.

### No significant relationship between visual gain and SIFI

In our final model, we assessed whether SIFI susceptibility was predicted by visual gain. We hypothesised that those for whom congruent auditory inputs benefit their visual judgements would also be influenced by audition even when incongruent to the visual information. Although the observed effects occurred in the hypothesised direction, this relationship failed to reach significance. One explanation of this is that if increased VTD is associated with stronger SIFI susceptibility^25^, as supported by our model 1a, and those with better VTD show reduced visual gains (model 2a), the combination of these effects may have weakened the hypothesised relationship between visual gain and SIFI susceptibility. Specifically, those with good VTD would show less gain (due to reduced reliance on audition) as well as stronger SIFI susceptibility. Thus, we cannot rule out that the counteracting relationships between VTD and SIFI and VTD and visual gain weaken the predicted relationship between visual gain and SIFI.

### Implications and future directions

The current findings suggest that age-related sensory change influences sensory integration in a manner consistent with reliability weighted accounts of multisensory perception^16–20^. This is the first time this model has been supported by data from a large sample of individuals aged over 50 years. Incorporating an assessment of multisensory integration within large-scale studies of ageing represents an important step forward in research on perceptual function. Nevertheless, large-scale data collection comes with some methodological cost. Primarily, due to time constraints within the TILDA health assessment, each condition was presented only twice. Thus, each participant’s score took on one of three discrete values (0, 0.5 or 1 proportion correct). This placed limitations on possible analyses (for example signal detection theory could not be implemented). Furthermore, it remains unknown whether two trials are sufficient to obtain a robust measure of multisensory integration. Nevertheless, the current findings show that factors expected to modulate multisensory integration under well-controlled laboratory paradigms also produced clear effects within our study. Our results therefore suggest the possibility that multisensory integration may be assessed with fewer trials, which has promising implications for research with populations in which prolonged testing is not desirable or possible.

Another possible limitation is that the SIFI is a novel task with which participants may have been unfamiliar at the time of testing. The SIFI literature with young adults suggests that task familiarity does not modulate SIFI perception, for example it is resistant to feedback training^45^. However training on related psychophysical tasks has been shown to modulate SIFI in older adults^32^. Future follow-up testing within TILDA will be able to inform whether task familiarity influenced SIFI performance in our cohort: we would expect that older adults show similar or stronger SIFI susceptibility compared with previous assessment, but not reduced susceptibility. In the unlikely event that performance improves, this would suggest that task familiarity influenced the measurement of multisensory integration in ageing, which is an important question to address. A further possibility is that in lab-based paradigms, more trials allows participants to familiarise themselves with the task. For example, the number of SOAs participants are exposed to can influence SIFI^34^. Future experimental work should therefore systematically examine the effect of number of trials upon SIFI susceptibility in ageing.

A recent review of audio-visual temporal perception in ageing^30^ suggested that unisensory function may account for the heterogeneous findings in the literature. Our results partially support this view, showing that the reliability of each sense may contribute towards individual differences in multisensory perception in ageing. Nevertheless, the relationship between age and SIFI susceptibility remained significant even when considering unisensory ability. There are several possible explanations for this and future research can help elucidate these influences. Firstly, ageing may result in a genuine change in the biological mechanisms underpinning multisensory function, although it has been suggested these mechanisms are maintained in ageing^14^. Secondly, integration may also be driven by factors other than the reliability of each sense. For example, it has been shown that the participant’s sex and cognitive factors account for some differences in SIFI susceptibility within the TILDA cohort^46^. Here we focused only on sensory measures that were immediately related to our hypotheses, however, a next step will be to consider the large number of cognitive assessments included within the TILDA study.

Finally, a reliance on prior knowledge may change with ageing. In Bayesian models of multisensory integration, prior knowledge guides integration, and older adults appear to be more reliant on these priors. For example, older adults show stronger pre-stimulus beta-power in the SIFI^21^, a phenomenon associated with increased SIFI susceptibility^47^ and the implementation of perceptual priors^48^. Behaviourally Chan, Connolly and Setti (2018) showed that older and younger adults differ in their SIFI susceptibility depending on the SOAs presented. Specifically, presenting more SOAs reduced the illusion in younger and older adults, suggesting both groups gathered information to update their perceptual priors. Thus, factors influencing the updating of perceptual priors may also result in age differences in integration.

An interesting direction for future research is how interventions aimed to improve unisensory function (i.e. hearing aids, glasses, cataract removal) might affect multisensory integration and whether this has additional, clinically relevant, benefits. It is likely that sensory interventions alter the reliability of sensory signals. Recent evidence has shown that mild hearing loss in ageing results in reduced SIFI susceptibility and hearing aid use may restore this to age-appropriate levels^29^. If improving unisensory function refines multisensory integration, this may have important implications for situations in which inefficient multisensory integration may have maladaptive consequences^6,31^.

## Conclusions

This study demonstrates the effect of age-related change in unisensory function on multisensory integration in the largest cohort of older adults tested to date. Age-related change in SIFI susceptibility was mediated by visual acuity and self-reported hearing, such that better scores in these measures predicted reduced and stronger SIFI susceptibility respectively, in line with reliability weighting models of multisensory perception. Unexpectedly, VTD improved with age and predicted reduced SIFI. This seemed to be consistent with self-reports of better vision relative to hearing with increasing age. Importantly, the relationship between age and SIFI susceptibility could not be explained via unisensory change alone, suggesting either genuine changes in multisensory mechanisms, a role of additional factors (such as cognition), or both. Better ATD and poorer visual acuity were associated with a greater use of auditory information to benefit visual temporal judgements (visual gain). Thus, older adults might be more likely to use audition to benefit vision if visual acuity is low and auditory temporal resolution high (consistent with reliability weighting). Our findings also indicate that subcomponents of vision (i.e. contrast and acuity) have differing effects on multisensory integration that may need further investigating. Future research should consider the impact that clinical interventions, aimed to improve unisensory function, might have upon multisensory integration and if this, in turn, has positive clinical outcomes.

## Method

### Participants

**Figure 4:**
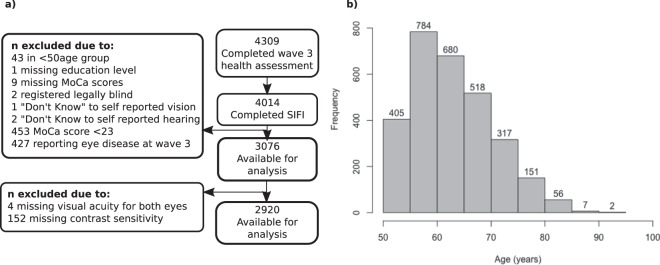
(**a**) Selection criteria for participants available from wave 3 of TILDA (**b**) distribution of ages in the sampled cohort.

Participants were drawn from wave 3 of the Irish Longitudinal Study on Ageing (TILDA), a population representative sample of individuals aged 50+ from the Republic of Ireland (Fig. 4). See^49^ for details of the sampling protocol. For pre-registration see https://osf.io/f4x8k/. (Note that in our pre-registration, we also planned to study change in sensory function between waves 1 and 3. These are provided within the Supplementary Material). The study was approved by the Trinity College Faculty of Health Sciences Ethics Committee, and testing protocols conformed with the Declaration of Helsinki. All participants provided written, informed consent when they first participated in the study (at wave 1) and consent was repeated at wave 3 (the focus of this study), both written and verbal. Respondents in all cases were provided with copies of their signed consent forms. Table 2 illustrates the demographic information of the retained sample.

**Table 2:** Demographic information for retained sample stratified by age group.

		Age (years)		
		50–64	65–74	>=75
Age	Mean	58.42	68.71	78.44
	SD	3.48	2.73	3.36
	Med.	58	68	78
Sex	F/M	998/745	482/423	113/159
MoCA	Mean	27.21	26.7	26.14
	SD	1.98	1.98	2.01
	Med.	27	27	26
Self-reported hearing	Mean	3.74	3.53	3.34
	SD	0.97	0.99	1.02
	Med.	4	4	3
Self-reported vision	Mean	3.71	3.7	3.76
	SD	0.85	0.83	0.84
	Med.	4	4	4
Visual acuity	Mean	97.87	95.39	93.73
	SD	8.11	8.53	7.97
	Med.	99	97	95
Contrast sensitivity	Mean	1.35	1.24	1.11
	SD	0.37	0.36	0.35
	Med.	1.43	1.26	1.15

Note: age was entered as continuous during modelling. For self-reported scores 1 = “Poor” and 5 = “Excellent, Med. = Median, MoCa = Montreal Cognitive Assessment.

### Material and measures

In addition to measures of multisensory function (outlined below), our analysis focused on the following measures of unisensory function; self-reported vision and self-reported hearing (1 = Poor to 5 = Excellent), visual acuity (assessed using the Early Treatment Diabetic Retinopathy Study (ETDRS) LogMAR chart, producing visual acuity scores as described by^50^ where larger scores indicate better acuity) and contrast sensitivity (assessed via an orientation discrimination paradigm, enabling calculation of area under the contrast sensitivity curve where larger scores indicate better performance). For further details on additional measures, please see Supplementary Material. We also used unimodal conditions from the SIFI paradigm to measure “Visual Temporal Discrimination” (VTD) and “Auditory Temporal Discrimination” (ATD) – outlined below.

#### The sound-induced flash illusion (SIFI)

The SIFI protocol was identical to that described by^46^. Participants attended a health assessment within TILDA for a total of approximately 3 hours. The SIFI procedure lasted approximately 6 minutes within this assessment. All assessments were carried out by trained nurses using a Standard Operating Procedure. If the participant usually wore glasses or hearing aids, they also wore them during the SIFI assessment. For the SIFI task, participants were seated in front of a computer and instructed to look at the fixation cross at the centre of the screen at the start of the task. Participants completed a short practice block before starting the experiment. The nurse sat near the participant and recorded the participant’s vocal responses to each trial by pressing corresponding number keys on a laptop. This was implemented to avoid problems for participants not accustomed with keyboards. A fixation cross marked the start of each trial and appeared for 1000 ms, to which the participant was instructed to fixate. The visual and/or auditory stimuli were then presented. Once a response was provided, the space bar had to be pressed to continue to the next trial.

All stimuli were presented via a laptop computer (DELL Latitude E6400 with Intel core 2 Duo CPU, 2 Gb RAM using Windows 7 Professional OS). The visual stimulus comprised a white disc, subtending a visual angle of approximately 1.5° and luminance of approximately 32fl, projected onto a black background and positioned 5 cm below the central fixation cross for 16 ms. The auditory beeps were brief bursts of 3500 Hz sounds (10 ms, 1 ms ramp), presented at approximately 80 dB via the inbuilt speakers in the laptop.

The four trial types used are outlined in Table 3. The unimodal auditory trials were presented first in a separate block, followed by the unimodal visual and multisensory (SIFI and non-illusory) trials, which were interleaved. This separation of testing blocks was necessary due to the different tasks involved: both visual unisensory and multisensory trials required participants to count the number of visual flashes, while the auditory block required participants to count the number of beeps.

**Table 3:** Parameters used for each condition.

Condition label		Number of beeps	Number of flashes	SOAs (ms)
Multisensory (SIFI)	2B1F	2	1	−230, −150, −70, 70, 150, 230
Multisensory (non Illusory)	2B2F	2	2	70, 150, 230
	1B1F	1	1	n/a
Unimodal visual	0B2F	0	2	70
Unimodal auditory	2B0F	2	0	70, 150, 230

SOA = Stimulus Onset Asynchrony, where negative/positive values indicate a beep preceded/followed the flash-beep pair. See text for details.

Each trial type was presented twice within a block and in random order across participants. Before testing, the practice phase was comprised of one trial from each of the following conditions: 2B1F and 2F2B (SOAs 70; 150; 230); 1B1F; 2F0B and auditory only trials (1B0F; 2B0F with SOAs 70; 150; 230).

#### Visual/auditory temporal discrimination

*Auditory Temporal Discrimination* (ATD) was defined as accuracy in detecting whether one or two beeps (with 70 ms SOA) were presented. Note that this differs from the definition of “Auditory temporal discrimination” reported in our pre-registration, in which we use a latent factor to represent auditory discrimination at three SOAs; 70, 150 and 230 ms. This change was implemented to simplify our model, enabling convergence and comparability. In parallel, *Visual Temporal Discrimination* (VTD) was defined as accuracy in identifying that two flashes (with 70 ms SOA) were presented. An SOA of 70 ms was used for measuring both ATD and VTD because, due to time constraints, the unimodal visual condition included only a 70 ms SOA, thus by using an SOA of 70 ms it would render performance on the ATD and VTD more comparable. Secondly, performance was very high for detecting two beeps at SOAs of both 230 ms (mean accuracy = 98%, SD = 13%) and 150 ms (mean accuracy = 95%, SD = 18%), compared to an SOA of 70 ms (mean accuracy = 57%, SD = 43%), thus at 70 ms SOA performance would not be attributed to ceiling effects.

### Data analysis

Statistical analyses were performed using the R statistical programming environment and run using R version 3.5.2^51^. Structural Equation Models (SEMs) were fit using the lavaan package for latent variable analysis^52^ using an Asymptotic Distribution Free estimation approach^53^ to relax the assumption of normality (implemented in lavaan using a Weighted Least Squares (WLS) estimator). Important outcome variables are defined below.

*SIFI susceptibility* was defined as lower proportion correct on multisensory, illusory trials. To reduce the dimensionality of our data, and simplify our models, SIFI susceptibility was captured as a latent variable derived from the measured accuracy in all 6 types of illusory 2B1F trials (i.e. with −230, −150, −70, 70, 150 and 230 ms SOAs).

*Visual gain* was calculated as:

1 $$Visual\,gain=Accuracy\,2B2F-Accuracy\,0B2F$$

in which both stimuli were presented with a 70 ms SOA. We refer to this score as “visual gain” because it reflects how much accuracy in detecting visual stimuli increases (or decreases) when visual stimuli are presented with, versus without, congruent sound.

The difference score between self-reported vision and hearing (i.e. *Self-reported vision − self-reported hearing*) was used to estimate the relative reliability of vision versus audition. This score therefore indicates how much more reliable participants rated their vision relative to audition.

Due to the known issues of implementing Chi-squared as a goodness-of-fit statistic with large sample sizes, goodness of fit was assessed using the Root Mean Square Error of Approximation (RMSEA). For models with a RMSEA > = 0.6 modification indices were computed to improve model fit^54^. The computed indices were then considered in turn and implemented if considered theoretically justifiable. For example, it has been shown that whilst decline in vision and hearing occur independently, participants generalise sensory loss across self-ratings of vision and hearing^3^. We therefore included covariance between self-reported vision and hearing to improve model fit where necessary.

## Supplementary information

Supplementary Information

## Data Availability

TILDA has a public database which includes unisensory measures included in the current analysis. The SIFI data is planned to be included in future releases of the TILDA dataset.
