# Supplementary material for: Age-related sensory decline mediates the Sound-Induced Flash Illusion: Evidence for reliability weighting models of multisensory perception
Source: Sci Rep. 2019 Dec 18;9:19347. doi: 10.1038/s41598-019-55901-5 (PMC6920348; doi:10.1038/s41598-019-55901-5)
Supplement: Supplementary file 1 — Supplementary Information [file 41598_2019_55901_MOESM1_ESM.pdf]

SUPPLEMENTARY MATERIAL

Title: Age-related sensory decline mediates the Sound-Induced Flash Illusion: Evidence for reliability weighting models of multisensory perception

Authors: Rebecca J. Hirst[1,2], Annalisa Setti[2,3], Rose Anne Kenny[2,4], Fiona N. Newell[1]

[1] School of Psychology and Institute of Neuroscience, Trinity College Dublin, Ireland

[2] The Irish Longitudinal Study on Ageing, Trinity College Dublin, Ireland

[3] School of Applied Psychology, University College Cork, Ireland

[4] Mercer Institute for Successful Ageing, St. James Hospital, Dublin, Ireland

Corresponding author:

Rebecca Hirst, hirstr@tcd.ie

Institute of Neuroscience

Trinity College Dublin

Dublin, Ireland

Content

S1 Outline of sensory measures

S2 Tables showing results of main analyses

Table S1: Model 1a – sensory function in relation to age and SIFI

Table S2: Model 1b – relative reliability in relation to age and SIFI

Table S3: Model 2a – sensory function in relation to age and Visual gain

Table S4: Model 2b – relative reliability in relation to age and Visual gain

Table S5: Model 3 – Visual gain in relation to SIFI

S3 Tables showing results for additional analysis of four-year change in sensory function relative to multisensory integration

Figure S1 – selection of participants for supplementary analysis

Table S6: T-tests comparing sensory function at wave 1 versus wave 3.

Table S7: Model 4a – change in sensory function in relation to age and SIFI

Table S8: Model 4b – change in relative reliability in relation to age and SIFI

Table S9: Model 5a – change in sensory function in relation to age and Visual gain

Table S10: Model 5b - change in relative reliability in relation to age and Visual gain

S1. Outline of sensory measures

S1.1 Self-reported vision and hearing

As part of a Computer Assisted Personal Interview (CAPI) participants were asked *"Is your eyesight (using glasses or contact lenses if you use them)… Excellent, Very good, Good, Fair or Poor?"* and *"Is your hearing (with or without a hearing aid)… Excellent, Very good, Good, Fair or Poor?"*. For vision, participants could also report if they were registered as legally blind. For both questions, participants were also given the option to report if they did not know (participants registered as legally blind or who responded "Don't know" were excluded from our analyses – Figure 1). For our analysis, scores were coded from 1 = Poor to 5 = Excellent. Thus, higher scores indicated better self-reported ability.

S1.2 Visual Acuity Scores

Visual acuity was measured using the Early Treatment Diabetic Retinopathy Study (ETDRS) LogMAR chart. This required participants to identify high contrast letters of decreasing size at a viewing distance of 4m. Visual acuity was assessed in both eyes and the eye giving the best acuity score was used for analysis. For statistical purposes, and to facilitate interpretation of our models, LogMAR measures were recoded to a visual acuity score (as in Donoghue et al., 2013), using the formula: VAS = 100 – 50 x LogMAR, so that a VAS of 100 represents a LogMAR score of 0 (20/20 vision), and higher scores therefore indicate better acuity.

S1.3 Contrast sensitivity

Contrast sensitivity was measured in the eye yielding the best visual acuity. Participants were presented with grating stimuli set to 5 levels of spatial frequency (1.5, 3, 6, 12 and 18 cycles per degree). Each spatial frequency was presented at 9 decreasing levels of contrast (.15 log unit or 50% loss of contrast between each stimulus). Participants were asked to judge whether the grating was oriented to the left (15°), right (-15°) or upright (0°). The threshold value for

each spatial frequency corresponds to the contrast of the last grating that the participant was able to identify. The 5 threshold values obtained for each participant were used to create individual contrast sensitivity functions by plotting log10 (spatial frequency) against log10 (contrast sensitivity). For some participants, it was only possible to derive thresholds for very few data points (i.e. 1 or 2). For this reason, a curve fitting approach was not appropriate, as curves would likely show a poor fit to the data. In cases where it was not possible to obtain a contrast sensitivity measure, within an individual at a specific spatial frequency, these values were set to 0 for plotting, indicating that the participant did not show measurable sensitivity at this spatial frequency. The trapezoid integration method was then used to derive the area under the data points in MATLAB. Thus, the measure of contrast sensitivity we use corresponds to the area under the contrast sensitivity curve, indicating each individual's functional range (across spatial frequencies), with larger values representing a larger functional range.

S2. Tables showing results of main analyses

**Model 1a**								
		Estimate	SE	z	P(>|z|)	Std.lv	Std.all	R squared
Factor loading onto "SIFI susceptibility"								
SOA	-230	1				0.292	0.753	
	-150	1.131	0.028	40.477	<.001	0.33	0.845	
	-70	0.877	0.027	32.694	<.001	0.256	0.584	
	70	0.92	0.027	34.533	<.001	0.268	0.613	
	150	1.284	0.031	40.792	<.001	0.375	0.879	
	230	1.143	0.023	48.889	<.001	0.334	0.766	
Regression coefficients								
Age →	Visual acuity	-0.222	0.02	-10.863	<.001	-0.222	-0.202	
	Contrast sensitivity	-0.011	0.001	-12.492	<.001	-0.011	-0.229	
	Self-reported vision	-0.002	0.002	-0.796	0.426	-0.002	-0.015	
	0B2F	0.005	0.001	5.538	<.001	0.005	0.103	

	2B0F	-0.001	0.001	-0.931	0.352	-0.001	-0.017	
	1B1F	-0.001	0	-1.739	0.082	-0.001	-0.051	
	Self-reported hearing	-0.022	0.002	-9.179	<.001	-0.022	-0.163	
→SIFI	Age	-0.006	0.001	-7.185	<.001	-0.02	-0.145	
	Visual acuity	0.002	0.001	2.495	0.013	0.006	0.051	
	Contrast sensitivity	-0.023	0.017	-1.359	0.174	-0.078	-0.028	
	Self-reported vision	0.016	0.007	2.197	0.028	0.053	0.044	
	0B2F	-0.079	0.017	-4.643	<.001	-0.272	-0.088	
	1B1F	0.222	0.043	5.228	<.001	0.761	0.075	
	2B0F	0.032	0.013	2.49	0.013	0.11	0.046	
	Self-reported hearing	-0.02	0.006	-3.411	0.001	-0.068	-0.068	
Covariance's								
Visual acuity	Contrast sensitivity	1.05	0.065	16.092	<.001	1.05	0.378	
Self-reported vision	Self-reported hearing (MI)	0.241	0.015	15.866	<.001	0.241	0.296	
-230	230	0.012	0.002	4.679	<.001	0.012	0.163	
-150	150	-0.009	0.002	-3.89	<.001	-0.009	-0.219	
-70	70	0.062	0.003	20.853	<.001	0.062	0.5	
0B2F	1B1F	-0.001	0.001	-1.24	0.215	-0.001	-0.023	
Variances								
	-230	0.065	0.003	24.368	<.001	0.065	0.432	0.568
	-150	0.044	0.003	16.31	<.001	0.044	0.287	0.713
	-70	0.127	0.003	40.021	<.001	0.127	0.659	0.341
	70	0.119	0.003	38.737	<.001	0.119	0.624	0.376
	150	0.041	0.003	13.487	<.001	0.041	0.228	0.772
	230	0.078	0.003	23.109	<.001	0.078	0.413	0.587
	Self-reported vision	0.692	0.016	42.875	<.001	0.692	1	0
	VAS	61.808	2.989	20.68	<.001	61.808	0.959	0.041
	AUC	0.125	0.004	30.604	<.001	0.125	0.948	0.052
	0B2F	0.103	0.003	35.056	<.001	0.103	0.989	0.011
	2B0F	0.177	0.002	83.36	<.001	0.177	1	0
	1B1F	0.01	0.001	8.22	<.001	0.01	0.997	0.003
	Self-reported hearing	0.959	0.02	46.972	<.001	0.959	0.973	0.027
	SIFI susceptibility	0.081	0.003	23.341	<.001	0.954	0.954	0.046
	Age	53.409	1.378	38.753	<.001	53.409	1	
**Mediation analysis**								
Direct effect	Age → SIFI	-0.006	0.001	-7.185	<.001	-0.02	-0.145	
Indirect effects	Age→0B2F →SIFI	0	0	-3.634	<.001	-0.001	-0.009	
	Age→ Contrast sensitivity→SIFI	0	0	1.354	0.176	0.001	0.006	

	Age→ Visual actuity →SIFI	0	0	-2.461	0.014	-0.001	-0.01	
	Age→ Self-reported vision →SIFI	0	0	-0.755	0.45	0	-0.001	
	Age→ self-reported hearing →SIFI	0	0	3.184	0.001	0.002	0.011	
	Age→ 2B0F→SIFI	0	0	-0.879	0.379	0	-0.001	
	Age→ 1B1F→SIFI	0	0	-1.841	0.066	-0.001	-0.004	
Total effects	Age→0B2F →SIFI	-0.006	0.001	-7.684	<.001	-0.021	-0.154	
	Age→ Contrast sensitivity→SIFI	-0.006	0.001	-6.948	<.001	-0.019	-0.138	
	Age→ Visual acuity →SIFI	-0.006	0.001	-7.693	<.001	-0.021	-0.155	
	Age→ Self-reported vision	-0.006	0.001	-7.2	<.001	-0.02	-0.145	
	Age→ Self-reported Hearing	-0.005	0.001	-6.687	<.001	-0.018	-0.133	
	Age→ 2B0F→SIFI	-0.006	0.001	-7.217	<.001	-0.02	-0.145	
	Age→ 1B1F→SIFI	-0.006	0.001	-7.326	<.001	-0.02	-0.148	

Table S1. Output parameters for model 1a. Estimate = parameter estimate, SE = Standard Error of the parameter estimate, Std.lv = Standardized latent variables, Std.all = Completely standardized coefficients. [MI] indicates that this parameter was added following inspection of modification indices. RMSEA for this model = .051, CI = [.047, .055], *pclose* = .356. 2B0F = 2-beep accuracy (ATD), 0B2F = 2-flash accuracy (VTD)

**Model 1b**								
		Estimate	SE	z	P(>|z|)	Std.lv	Std.all	R squared
Factor loading onto "SIFI susceptibility"								
SOA	-230	1				0.277	0.714	
	-150	1.219	0.035	34.415	<.001	0.336	0.861	
	-70	0.819	0.028	28.832	<.001	0.226	0.512	
	70	0.871	0.028	31.328	<.001	0.24	0.547	
	150	1.397	0.041	34.415	<.001	0.385	0.905	
	230	1.14	0.025	45.503	<.001	0.314	0.719	
Regression coefficients								
Age →	Subjective reliability	0.021	0.003	7.854	<.001	0.021	0.141	
→SIFI	Age	-0.006	0.001	-7.844	<.001	-0.021	-0.155	
	Subjective reliability	0.017	0.005	3.524	<.001	0.062	0.069	
Covariance's								
-230	230	0.022	0.003	8.107	<.001	0.022	0.265	
-150	150	-0.02	0.003	-6.757	<.001	-0.02	-0.544	
-70	70	0.078	0.003	25.505	<.001	0.078	0.564	
Variances								
	-230	0.073	0.003	26.331	<.001	0.073	0.49	0.51
	-150	0.039	0.003	12.532	<.001	0.039	0.258	0.742
	-70	0.143	0.003	45.589	<.001	0.143	0.738	0.262
	70	0.135	0.003	43.99	<.001	0.135	0.701	0.299
,	150	0.033	0.004	8.889	<.001	0.033	0.182	0.818
	230	0.092	0.004	25.551	<.001	0.092	0.484	0.516
	Subjective reliability	1.189	0.034	35.363	<.001	1.189	0.98	0.02

	SIFI	0.074	0.004	20.743	<.001	0.974	0.974	0.026
	Age	53.653	1.394	38.502	<.001	53.653	1	
**Mediation analysis**								
Direct effect	Age → SIFI	-0.006	0.001	-7.844	<.001	-0.021	-0.155	
Indirect effects	Age→ Subjective reliability →SIFI	0	0	3.197	0.001	0.001	0.01	
Total effects	Age→ Subjective reliability →SIFI	-0.005	0.001	-7.376	<.001	-0.02	-0.145	

Table S2. Output parameters for model 1b. Estimate = parameter estimate, SE = Standard Error of the parameter estimate, Std.lv = Standardized latent variables, Std.all = Completely standardized coefficients. RMSEA for this model = .05 CI = [.042, .058], *pclose* = .474.

**Model 2a**								
		Estimate	SE	z	P(>|z|)	Std.lv	Std.all	R squared
Regression coefficients								
Age →	Visual acuity	-0.222	0.02	-11.013	<.001	-0.222	-0.205	
	Contrast sensitivity	-0.012	0.001	-13.47	<.001	-0.012	-0.242	
	Self-reported vision	-0.001	0.002	-0.274	0.784	-0.001	-0.005	
	0B2F	0.001	0.001	1.443	0.149	0.001	0.026	
	2B0F	-0.004	0.001	-3.857	<.001	-0.004	-0.072	
	Self-reported hearing	-0.022	0.002	-9.098	<.001	-0.022	-0.164	
	1B1F	-0.001	0	-1.318	0.188	-0.001	-0.035	
→Visual gain	Age	0	0.001	-0.105	0.916	0	-0.001	
	Visual acuity	-0.003	0.001	-2.999	0.003	-0.003	-0.045	
	Contrast sensitivity	0.052	0.02	2.605	0.009	0.052	0.038	
	Self-reported vision	-0.011	0.009	-1.263	0.207	-0.011	-0.018	
	0B2F	-0.935	0.02	-46.039	<.001	-0.935	-0.615	
	2B0F	0.339	0.016	21.034	<.001	0.339	0.283	
	Self-reported hearing	0.011	0.008	1.441	0.149	0.011	0.021	
	1B1F	-0.017	0.071	-0.246	0.806	-0.017	-0.004	
Covariance's								
Visual acuity	Contrast sensitivity	1.072	0.067	16.012	<.001	1.072	0.378	
Self-reported vision	Self-reported hearing [(MI)]	0.245	0.015	15.904	<.001	0.245	0.301	

0B2F	1B1F	0	0.001	-0.142	0.887	0	-0.002	
Variances								
	Self-reported vision	0.697	0.016	42.684	<.001	0.697	1	0
	Visual acuity	62.118	3.072	20.223	<.001	62.118	0.958	0.042
	Contrast sensitivity	0.13	0.004	31.071	<.001	0.13	0.942	0.058
	0B2F	0.113	0.003	36.006	<.001	0.113	0.999	0.001
	2B0F	0.181	0.002	81.691	<.001	0.181	0.995	0.005
	Self-reported hearing	0.951	0.021	45.957	<.001	0.951	0.973	0.027
	1B1F	0.012	0.001	8.124	<.001	0.012	0.999	0.001
	Visual gain	0.14	0.003	54.457	<.001	0.14	0.538	0.462
	Age	55.238	1.405	39.311	<.001	55.238	1	
Direct effect	Age → Visual gain	0	0.001	-0.105	0.916	0	-0.001	
Indirect effects	Age→0B2F →Visual gain	-0.001	0.001	-1.442	0.149	-0.001	-0.016	
	Age→ Contrast sensitivity → Visual gain	-0.001	0	-2.548	0.011	-0.001	-0.009	
	Age→ Visual acuity → Visual gain	0.001	0	2.904	0.004	0.001	0.009	
	Age→ Self-reported vision → Visual gain	0	0	0.268	0.789	0	0	
	Age→ Self-reported hearing → Gain	0	0	-1.422	0.155	0	-0.003	
	Age→ 2B0F→ Visual gain	-0.001	0	-3.789	<.001	-0.001	-0.02	
	Age→ 1B1F→ Visual gain	0	0	0.241	0.809	0	0	
Total effects	Age→0B2F → Visual gain	-0.001	0.001	-0.991	0.322	-0.001	-0.018	
	Age→ Contrast sensitivity → Visual	-0.001	0.001	-0.771	0.441	-0.001	-0.011	

	gain							
	Age→ Visual acuity → Visual gain	0.001	0.001	0.554	0.58	0.001	0.008	
	Age→ Self-reported vision → Visual gain	0	0.001	-0.098	0.922	0	-0.001	
	Age→ Self-reported Hearing → Visual gain	0	0.001	-0.361	0.718	0	-0.005	
	Age→ 2B0F→ Visual gain	-0.001	0.001	-1.444	0.149	-0.001	-0.022	
	Age→ 1B1F→ Visual gain	0	0.001	-0.096	0.924	0	-0.001	

Table S3. Output parameters for model 2a. Estimate = parameter estimate, SE = Standard Error of the parameter estimate, Std.lv = Standardized latent variables, Std.all = Completely standardized coefficients. [MI] indicates that this parameter was added following inspection of modification indices. RMSEA for this model = .042, CI = [.034, .049], *pclose* = .960.

Model 2b

		Estimate	SE	z	P(>|z|)	Std.lv	Std.all	R squared
Regression coefficients								
Age →	Subjective reliability	0.021	0.003	7.964	<.001	0.021	0.143	
→Visual gain	Age	-0.002	0.001	-1.873	0.061	-0.002	-0.035	
	Subjective reliability	-0.017	0.008	-2.021	0.043	-0.017	-0.038	
Variances								
	Subjective reliability	0.253	0.006	42.909	<.001	0.253	0.997	0.003
	Visual gain	1.196	0.034	35.377	<.001	1.196	0.979	0.021
	Age	55.224	1.412	39.1	<.001	55.224	1	
**Mediation analysis**								
Direct effect	Age → Visual gain	-0.002	0.001	-1.873	0.061	-0.002	-0.035	
Indirect effects	Age→ Subjective reliability → Visual gain	0	0	-1.963	0.05	0	-0.005	
Total effects	Age→ Subjective reliability → Visual gain	-0.003	0.001	-2.179	0.029	-0.003	-0.04	

Table S4. Output parameters for model 1b. Estimate = parameter estimate, SE = Standard Error of the parameter estimate, Std.lv = Standardized latent variables, Std.all = Completely standardized coefficients. "Subjective reliability" corresponds to the Self-reported vision – Self-reported hearing difference score.

Model 3

		Estimate	SE	z	P(>|z|)	Std.lv	Std.all	R squared
Factor loadings onto "SIFI susceptibility"								

SOA	-230	1				0.268	0.695	
	-150	1.276	0.039	32.464	<.001	0.342	0.876	
	-70	0.825	0.029	28.641	<.001	0.221	0.501	
	70	0.879	0.028	31.192	<.001	0.236	0.537	
	150	1.465	0.045	32.208	<.001	0.393	0.922	
	230	1.122	0.025	44.324	<.001	0.301	0.686	
Regression coefficients								
→SIFI	Visual gain	-0.016	0.01	-1.698	0.09	-0.06	-0.03	
Covariance's								
-230	230	0.028	0.003	10	<.001	0.028	0.312	
-150	150	-0.025	0.003	-7.771	<.001	-0.025	-0.798	
-70	70	0.081	0.003	26.327	<.001	0.081	0.571	
Variances								
	-230	0.077	0.003	27.398	<.001	0.077	0.517	0.483
	-150	0.036	0.003	10.532	<.001	0.036	0.233	0.767
	-70	0.146	0.003	46.775	<.001	0.146	0.749	0.251
	70	0.137	0.003	44.898	<.001	0.137	0.711	0.289
	150	0.027	0.004	6.688	<.001	0.027	0.15	0.85
	230	0.102	0.004	27.328	<.001	0.102	0.53	0.47
	SIFI	0.072	0.004	19.748	<.001	0.999	0.999	0.001
	Visual gain	0.252	0.006	43.884	<.001	0.252	1	

Table S5. Output parameters for model 1b. Estimate = parameter estimate, SE = Standard Error of the parameter estimate, Std.lv = Standardized latent variables, Std.all = Completely standardized coefficients. RMSEA for this model = .042 CI = [0.33, .052], *pclose* = .896.

S3. Additional analysis of four-year change in sensory function relative to multisensory integration

Within our initial analysis plan (for preregistration see https://osf.io/f4x8k/?view_only=d8b69d511dc4418d90530af549be3b1c) we intended to study multisensory integration in relation to change in sensory ability from wave 1 to wave 3 of TILDA, in addition to sensory ability at the time of SIFI (as presented within our main manuscript). However, despite significant differences in sensory measures between wave 1 and 3 (see Table S6), we did not find an effect of change in sensory function upon SIFI susceptibility in ageing. One explanation of this might be that the extent of sensory decline between waves 1 and 3 was not large enough to observe change effects. Indeed, the TILDA study recently adjusted its protocol to shift scheduled health assessments from wave 5 to wave 6 in order to increase observed age-related changes. For this reason, we include the results of these analyses as Supplementary Material for the interested reader.

	Wave 1		Wave 3		*t*	*p*
	M	SD	M	SD		
**Visual acuity**	98.59	8.22	96.8	8.31	10.72	<.001
**Contrast sensitivity**	1.35	.339	1.30	.376	6.668	<.001
**Self-reported vision**	3.84	.869	3.71	.848	6.034	<.001
**Self-reported hearing**	3.84	1.03	3.64	.989	9.750	<.001
**Subjective reliability**	.002	1.14	.074	1.12	-2.777	.005

Table S6. Results of paired sample t-tests comparing sensory abilities at wave 1 versus wave 3. Df for all = 2420. M =

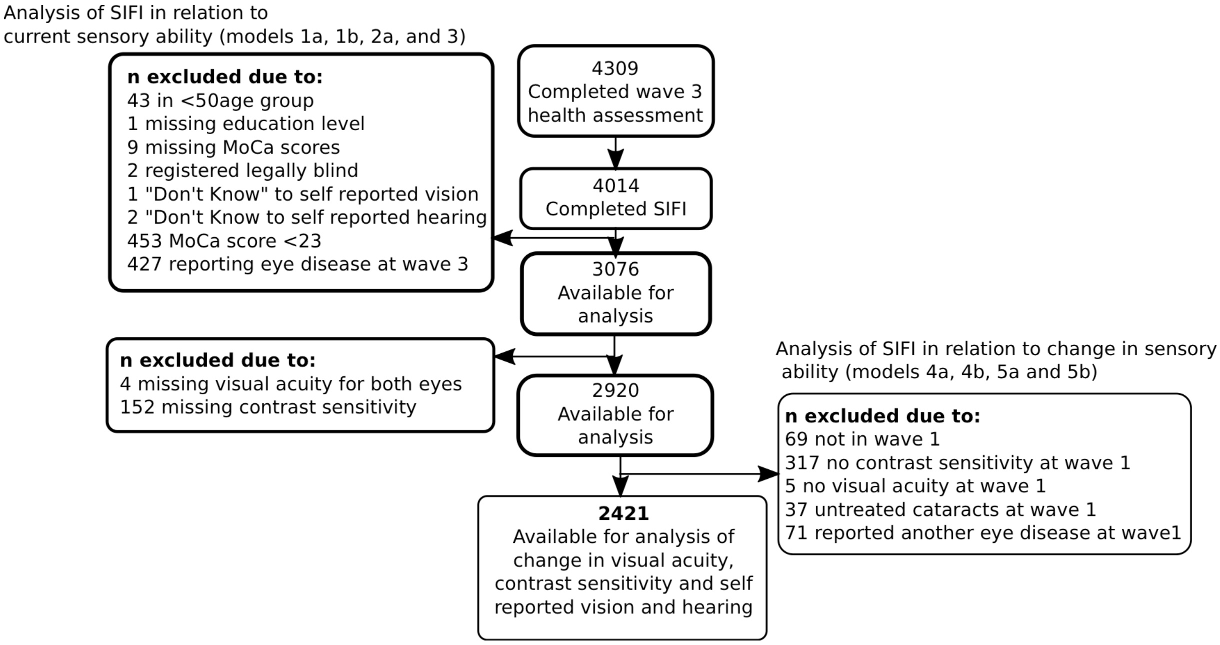

Figure S1. Participant selection for supplementary analysis of change in sensory function.

Figure S1 shows the selection of participants for analysis of change scores. To be included in this analysis, participants must have taken part in both wave 1 and wave 3 of TILDA. Change scores were calculated as the difference between the measure of sensory ability at wave 1 versus at wave 3. Thus, higher scores indicate better ability at wave 1 (i.e. a larger amount of change).

Model 4a

Model 4a aimed to assess whether change in sensory function over time mediated the relationship between age and SIFI. This model was identical to model 1a except that measures of sensory function were converted to change scores from wave 1 to wave 3. VTD, ATD and performance in the 1B1F condition were removed as predictors (because these were not available for wave 1). As shown in Table S7, no change scores mediated the relationship between age and SIFI.

**Model 4a**								
		Estimate	SE	z	P(>|z|)	Std.lv	Std.all	R squared
Factor loading onto "SIFI susceptibility"								
SOA	-230	1				0.279	0.723	
	-150	1.217	0.038	31.73	<.001	0.339	0.867	
	-70	0.83	0.031	27.079	<.001	0.231	0.526	
	70	0.852	0.03	28.638	<.001	0.237	0.543	
	150	1.37	0.043	32.103	<.001	0.382	0.903	
	230	1.105	0.027	41.468	<.001	0.308	0.709	
Regression coefficients								
Age →	Visual acuity (change)	0.006	0.023	0.263	0.793	0.006	0.005	
	Contrast sensitivity (change)	0.004	0.001	3.955	<.001	0.004	0.08	
	Self-reported vision (change)	0.006	0.003	2.145	0.032	0.006	0.045	
	Self-reported hearing (change)	0.001	0.003	0.266	0.79	0.001	0.005	
→SIFI	Age	-0.006	0.001	-7.065	<.001	-0.021	-0.151	
	Visual acuity (change)	0	0.001	0.727	0.467	0.002	0.015	
	Contrast sensitivity (change)	0.003	0.016	0.189	0.85	0.011	0.004	
	Self-reported vision (change)	-0.003	0.006	-0.457	0.648	-0.01	-0.01	
	Self-reported hearing (change)	0.001	0.006	0.154	0.877	0.004	0.003	
Covariance's								
VAS (change)	Contrast sensitivity (change)	0.716	0.076	9.465	<.001	0.716	0.23	
-230	230	0.023	0.003	7.916	<.001	0.023	0.28	
-150	150	-0.02	0.003	-6.325	<.001	-0.02	-0.563	
-70	70	0.077	0.003	23.239	<.001	0.077	0.562	
Variances								
	-230	0.071	0.003	23.873	<.001	0.071	0.477	0.523
	-250	0.038	0.003	10.974	<.001	0.038	0.248	0.752
	-70	0.14	0.003	40.383	<.001	0.14	0.724	0.276
	70	0.135	0.003	40.487	<.001	0.135	0.705	0.295
	150	0.033	0.004	8.447	<.001	0.033	0.185	0.815
	230	0.094	0.004	23.953	<.001	0.094	0.497	0.503
	Self-reported vision	0.936	0.03	31.538	<.001	0.936	0.998	0.002

	(change)							
	Visual acuity (change)	71.398	3.085	23.145	<.001	71.398	1	0
	Contrast sensitivity (change)	0.136	0.006	23.687	<.001	0.136	0.994	0.006
	Self-reported hearing (change)	0.916	0.029	31.215	<.001	0.916	1	0
	SIFI	0.076	0.004	19.075	<.001	0.977	0.977	0.023
	Age	52.544	1.491	35.236	<.001	52.544	1	
**Mediation analysis**								
Direct effect	Age → SIFI	-0.006	0.001	-7.065	<.001	-0.021	-0.151	
Indirect effects	Age→ Contrast sensitivity (change) →SIFI	0	0	-0.445	0.656	0	0	
	Age→ Visual acuity (change) →SIFI	0	0	0.246	0.805	0	0	
	Age→ Self-reported vision (change) →SIFI	0	0	0.189	0.85	0	0	
	Age→ Self-reported Hearing (change)→SIFI	0	0	0.132	0.895	0	0	
Total effects	Age→ Contrast sensitivity (change)→SIFI	-0.006	0.001	-7.066	<.001	-0.021	-0.151	
	Age→ Visual acuity (change)→ SIFI	-0.006	0.001	-7.083	<.001	-0.021	-0.151	
	Age→ Self-reported vision (change)→SIFI	-0.006	0.001	-7.06	<.001	-0.021	-0.151	
	Age→ Self-reported Hearing (change)→SIFI	-0.006	0.001	-7.098	<.001	-0.021	-0.151	

Table S7. Output parameters for model 1a. Estimate = parameter estimate, SE = Standard Error of the parameter estimate, Std.lv = Standardized latent variables, Std.all = Completely standardized coefficients. [MI] indicates that this parameter was added following inspection of modification indices. RMSEA for this model = .047, CI = [.042, .053], *pclose* = .762

Model 4b

Model 4b aimed to assess whether change in relative sensory reliability over time mediated the relationship between age and SIFI. This model was identical to model 1b except that change in subjective reliability (between wave 1 and wave 3) was considered a mediator. As shown in Table S8, no change scores mediated the relationship between age and SIFI.

**Model 4b**								
		Estimate	SE	z	P(>|z|)	Std.lv	Std.all	R squared
Factor loading onto “SIFI susceptibility”								
SOA	-230	1				0.277	0.722	
	-150	1.218	0.039	31.429	<.001	0.337	0.862	
	-70	0.825	0.031	26.67	<.001	0.229	0.52	
	70	0.86	0.03	28.416	<.001	0.238	0.545	

	150	1.38	0.043	31.786	<.001	0.382	0.902	
	230	1.122	0.027	40.994	<.001	0.311	0.713	
Regression coefficients								
Age →	Subjective reliability (change)	0.005	0.003	1.322	0.186	0.005	0.026	
→SIFI	Age	-0.006	0.001	-7.201	<.001	-0.021	-0.154	
	Subjective reliability (change)	-0.002	0.005	-0.38	0.704	-0.006	-0.008	
Covariance's								
-230	230	0.022	0.003	7.602	<.001	0.022	0.272	
-150	150	-0.019	0.003	-6.11	<.001	-0.019	-0.531	
-70	70	0.076	0.003	22.839	<.001	0.076	0.555	
Variances								
	-230	0.071	0.003	23.667	<.001	0.071	0.479	0.521
	-250	0.039	0.003	11.355	<.001	0.039	0.257	0.743
	-70	0.141	0.003	40.731	<.001	0.141	0.73	0.27
	70	0.134	0.003	39.94	<.001	0.134	0.703	0.297
,	150	0.034	0.004	8.495	<.001	0.034	0.187	0.813
	230	0.094	0.004	23.556	<.001	0.094	0.492	0.508
	Subjective reliability (change)	1.601	0.049	32.389	<.001	1.601	0.999	0.001
	SIFI	0.075	0.004	18.861	<.001	0.976	0.976	0.024
	Age	52.537	1.499	35.056	<.001	52.537	1	
**Mediation analysis**								
Direct effect	Age → SIFI	-0.006	0.001	-7.201	<.001	-0.021	-0.154	
Indirect effects	Age→ Subjective reliability (change)→SIFI	0	0	-0.365	0.715	0	0	
Total effects	Age→ Subjective reliability (change)→SIFI	-0.006	0.001	-7.215	<.001	-0.021	-0.154	

Table S8. Output parameters for model 1b. Estimate = parameter estimate, SE = Standard Error of the parameter estimate, Std.lv = Standardized latent variables, Std.all = Completely standardized coefficients. RMSEA for this model = .049 CI = [.04, .058], *pclose* = .568. "Subjective reliability" corresponds to the Self-reported vision – Self-reported hearing difference score.

Model 5a

Model 5a aimed to assess whether change in sensory function over time mediated the relationship between age and Visual gain. This model was identical to model 2a except that measures of sensory function were converted to change scores from wave 1 to wave 3. VTD and ATD were removed as predictors (because these were not available for wave1). As shown in Table S9, no change scores mediated the relationship between age and SIFI.

**Model 5a**								
		Estimate	SE	z	P(>|z|)	Std.lv	Std.all	R squared
Regression coefficients								
Age →	Visual acuity (change)	-0.006	0.023	-0.256	0.798	-0.006	-0.005	
	Contrast sensitivity (change)	0.004	0.001	4.298	<.001	0.004	0.086	
	Self-reported vision (change)	0.003	0.003	1.151	0.25	0.003	0.023	
	Self-reported hearing (change)	0	0.003	-0.103	0.918	0	-0.002	
→Visual gain	Age	-0.003	0.001	-1.939	0.053	-0.003	-0.04	
	Visual acuity (change)	0.002	0.001	1.328	0.184	0.002	0.028	
	Contrast sensitivity (change)	-0.03	0.028	-1.082	0.279	-0.03	-0.023	
	Self-reported vision (change)	0.002	0.01	0.184	0.854	0.002	0.004	
	Self-reported hearing (change)	-0.014	0.01	-1.332	0.183	-0.014	-0.027	
Covariance's								
VAS (change)	Contrast sensitivity (change)	0.727	0.077	9.413	<.001	0.727	0.23	
SR vision (change)	Self-reported hearing (change) [MI]	0.198	0.021	9.561	<.001	0.198	0.197	
Variances								
	Self-reported vision (change)	1.032	0.031	33.059	<.001	1.032	0.999	0.001
	Visual acuity (change)	71.033	3.163	22.458	<.001	71.033	1	0
	Contrast sensitivity (change)	0.14	0.006	23.773	<.001	0.14	0.993	0.007
	Self-reported hearing (change)	0.983	0.031	32.131	<.001	0.983	1	0
	Visual gain	0.253	0.006	39.423	<.001	0.253	0.997	0.003
	Age	53.999	1.513	35.694	<.001	53.999	1	
**Mediation analysis**								
Direct effect	Age → Visual gain	-0.003	0.001	-1.939	0.053	-0.003	-0.04	
Indirect effects	Age→ Contrast sensitivity (change) → Visual gain	0	0	0.181	0.856	0	0	
	Age→ Visual acuity (change) → Visual gain	0	0	-0.253	0.8	0	0	
	Age→ Self-reported vision (change) → Visual gain	0	0	-1.036	0.3	0	-0.002	
	Age→ Self-reported hearing (change)→ Visual gain	0	0	0.103	0.918	0	0	
Total effects	Age→ Contrast sensitivity (change)→ Visual gain	-0.003	0.001	-1.935	0.053	-0.003	-0.04	
	Age→ Visual acuity (change)→ Visual gain	-0.003	0.001	-2.034	0.042	-0.003	-0.042	
	Age→ Self-reported vision (change)→ Visual gain	-0.003	0.001	-1.943	0.052	-0.003	-0.04	
	Age→ Self-reported hearing (change)→ Visual gain	-0.003	0.001	-1.937	0.053	-0.003	-0.04	

Table S9. Output parameters for model 1a. Estimate = parameter estimate, SE = Standard Error of the parameter estimate, Std.lv = Standardized latent variables, Std.all = Completely standardized coefficients. [MI] indicates that this parameter was added following inspection of modification indices. RMSEA for this model = .004, CI = [0, .031], *pclose* = 1

Model 5b

Model 5b aimed to assess whether change in relative sensory reliability over time mediated the relationship between age and Visual gain. This model was identical to model 2b except that measures only subjective reliability scores were used, and this score was a difference score between relative reliability at wave 1 versus wave 3. As shown in Table S10, no change scores mediated the relationship between age and SIFI.

**Model 5b**								
		Estimate	SE	z	P(>|z|)	Std.lv	Std.all	R squared
Regression coefficients								
Age →	Subjective reliability (change)	0.004	0.003	1.048	0.294	0.004	0.021	
→Gain	Age	-0.003	0.001	-2.033	0.042	-0.003	-0.042	
	Subjective reliability (change)	0.008	0.008	1.032	0.302	0.008	0.021	
Variances								
	Subjective reliability (change)	1.621	0.05	32.646	<.001	1.621	1	0
	Visual gain	0.253	0.006	39.414	<.001	0.253	0.998	.002
	Age	53.892	1.514	35.585	<.001	53.892	1	
**Mediation analysis**								
Direct effect	Age → Visual gain	-0.003	0.001	-2.033	0.042	-0.003	-0.042	
Indirect effects	Age→ Subjective reliability (change)→ Visual gain	0	0	0.728	0.467	0	0	
Total effects	Age→ Subjective reliability (change)→ Visual gain	-0.003	0.001	-2.012	0.044	-0.003	-0.041	

Table S10. Output parameters for model 1b. Estimate = parameter estimate, SE = Standard Error of the parameter estimate, Std.lv = Standardized latent variables, Std.all = Completely standardized coefficients. "Subjective reliability" corresponds to the Self-reported vision – Self-reported hearing difference score.
